# A Tightly Integrated Navigation Method of SINS, DVL, and PS Based on RIMM in the Complex Underwater Environment

**DOI:** 10.3390/s22239479

**Published:** 2022-12-04

**Authors:** Huibao Yang, Xiujing Gao, Hongwu Huang, Bangshuai Li, Jiehong Jiang

**Affiliations:** 1 School of Aerospace Engineering, Xiamen University, Xiamen 361000, China; 2 Institute of Smart Marine and Engineering, Fujian University of Technology, Fuzhou 350118, China; 3 State Key Laboratory of Advanced Design and Manufacturing for the Vehicle Body, Hunan University, Changsha 410082, China

**Keywords:** autonomous underwater vehicle, the strap-down inertial navigation system, Doppler velocity log, the water-track model, the tightly integrated navigation, robust interacting multiple models

## Abstract

Navigation and positioning of autonomous underwater vehicles (AUVs) in the complex and changeable marine environment are crucial and challenging. For the positioning of AUVs, the integrated navigation of the strap-down inertial navigation system (SINS), Doppler velocity log (DVL), and pressure sensor (PS) has a common application. Nevertheless, in the complex underwater environment, the DVL performance is affected by the current and complex terrain environments. The outliers in sensor observations also have a substantial adverse effect on the AUV positioning accuracy. To address these issues, in this paper, a novel tightly integrated navigation model of the SINS, DVL, and PS is established. In contrast to the traditional SINS, DVL, and PS tightly integrated navigation methods, the proposed method in this paper is based on the velocity variation of the DVL beam by applying the DVL bottom-track and water-track models. Furthermore, a new robust interacting multiple models (RIMM) information fusion algorithm is proposed. In this algorithm, DVL beam anomaly is detected, and the Markov transfer probability matrix is accordingly updated to enable quick model matching. By simulating the motion of the AUV in a complex underwater environment, we also compare the performance of the traditional loosely integrated navigation (TLIN) model, the tightly integrated navigation (TTIN) model, and the IMM algorithm. The simulation results show that because of the PS, the velocity and height in the up-change amplitude of the four algorithms are small. Compared with the TLIN algorithm in terms of maximum deviation of latitude and longitude, the RIMM algorithm also improves the accuracy by 39.1243 m and 26.4364 m, respectively. Furthermore, compared with the TTIN algorithm, the RIMM algorithm improves latitude and longitude accuracy by 1.8913 m and 11.8274 m, respectively. A comparison with IMM also shows that RIMM improves the accuracy of latitude and longitude by 1.1506 m and 7.2301 m, respectively. The results confirm that the proposed algorithm suppresses the observed noise and outliers of DVL and further achieves quick conversion between different DVL models while making full use of the effective information of the DVL beams. The proposed method also improves the navigation accuracy of AUVs in complex underwater environments.

## 1. Introduction

Autonomous underwater vehicles (AUVs) have a wide range of applications, such as ocean pollutant monitoring [1], marine biology exploration [2], and pipeline inspection [3]. Such applications have an annual potential market of billions of dollars [4]. The underwater vehicle navigation system is a vital enabler for such applications by providing information such as position, velocity, and attitude [5]. However, the accuracy of the position information depends on the AUV’s working environment.

In the underwater environment, the global positioning system (GPS) is unavailable. As an alternative, the strap-down inertial navigation system (SINS) is often considered an essential part of the navigation system. Nevertheless, SINS is affected by the inherent drift error of the inertial sensors; hence, it is unable to provide long-term high positioning accuracy. The error accumulation in SINS is partly addressed by using the Doppler velocity log (DVL) as an auxiliary sensor. Therefore, SINS and DVL integrated navigation systems are commonly used for AUVs.

In the complex underwater environment, the SINS/DVL integrated navigation system needs to face several navigation challenges. To improve the accuracy of the integrated navigation system, a variety of filter models and algorithms have been proposed. The Kalman filter (KF) is a well-known technique for integrated navigation applications; see, e.g., [6,7,8,9]. Nevertheless, the traditional Kalman filter can be applied to linear systems, whereas navigation systems often demonstrate nonlinear behaviors. To address this issue, the extended Kalman filter (EKF) and the unscented Kalman filter (UKF) are used in the integrated navigation system [10,11,12]. Karimi et al. compared EKF and UKF algorithms for the inertial navigation system (INS) and DVL integrated system. Their investigations showed that the EKF results are closer to the actual values than those of the UKF [13]. Xing et al. proposed an extended Kalman filter (EKF) to synthesize the multi-source information from an inertial measurement unit (IMU), optical flow, pressure sensor, and ArUco markers. The proposed method enables the robot to obtain highly accurate positioning [14]. The accuracy of both EKF and UKF is also influenced by other factors such as filter model and noise characteristics.

To further improve the accuracy, an adaptive Kalman filter (AKF) was designed for the SINS/DVL integrated navigation system [15]. Gao et al. proposed an AKF algorithm that has a recursive noise estimator [16]. Huang et al. also devised an improved variational AKF based on the expectation-maximization (EM) algorithm (VAKFEM) [17]. The results confirm that the proposed AKF improves the estimation accuracy effectively and that the AKF is robust in the presence of vigorous maneuvers and rough sea conditions. Combining deep learning techniques with KF is also considered in the literature to improve navigation system stability. For instance, Li et al. built a nonlinear autoregressive with an exogenous input model subject to the availability of DVL. They then showed that this model could predict the output of DVL [18]. For cases where the DVL information is missing, Zhu et al. proposed a hybrid prediction method by combining the long short-term memory neural network (LSTM) and machine-learning-assisted adaptive filtering [19]. The above study is, however, based on the loosely integrated navigation model, which cannot make effective use of sensor data. The loose system structure can achieve good data fusion results in a less disturbed underwater environment.

DVL is an active sonar system; hence, it is easily affected in complex underwater environments. There exist marine creatures, large distance trenches, and powerful sound-absorbing materials in the underwater environment. These factors affect the accuracy of the DVL beam measurements. Furthermore, water velocity variation strongly affects the SINS and DVL integrated navigation system.

To fully exploit the valid information in the sensor data fusion process, tightly integrated navigation models are also considered. Liu et al. [20] built a tight navigation model that involves two types of DVL with four beams in the Janus structure. Additionally, Wang et al. [21] built a tightly integrated navigation model based on the 3-D velocity of DVL. Shede et al. [22] built a tightly integrated navigation system based on dual adaptive factors, which suppress the DVL outliers.

To handle the measurement noise variance of each DVL beam individually, Jin et al. [23] proposed a tightly coupled method in which an adaptive Kalman filter was utilized to dynamically estimate the observation noise. Xu et al. [24] applied the statistical similarity measure (SSM) to quantify the similarity between two random vectors of DVL. They then built a cost function to avoid the loss of normal measurement information. Yona et al. [25] also applied deep learning algorithms for the compensation of DVL beam outliers. These studies used the normal beam information of DVL through tightly integrated models. Nevertheless, these models often use the bottom-track velocity measurement of DVL, which is a simplification of the water-track model of DVL. Therefore, in complex underwater environments, these techniques have a limited detection range for DVL. A single observation variance matrix is unsuitable for the integrated navigation system during long voyages.

Since a single model is unable to characterize the complex motion environment, an interacting multiple models (IMM) algorithm is proposed for underwater navigation. IMM was first applied to target-tracking missions to autonomously integrate multiple models [26,27]. It has then been used in positioning applications to describe the uncertainty of the system model and statistics characteristic of observation noise. Yao et al. [28] applied an IMM-aided zero velocity update (ZUPT) technology for an INS/DVL integrated navigation system to mitigate the navigation error during the pure INS mode. Further, Yao et al. [29] proposed the IMM-UKF-aided SINS and ultra-short baseline (USBL) calibration solution. It was shown that the proposed solution could maintain its robustness when the quality of observation changed. To further enhance INS/DVL navigation system performance in the complex underwater environment, a hybrid interacting multiple models (HIMM) algorithm was proposed in [30], which includes both bottom-track and water-track velocity measurements of DVL. This method effectively limits DVL’s bottom-track outages. Zhu and He [31] proposed a robust IMM-KF for INS/DVL integrated navigation. However, their proposed method uses two fixed measurement covariance matrices, which might not be able to fully cover the actual model. To address this issue, Zhang et al. [32] proposed an improved interacting multiple model-unscented Kalman filter (IIMM-UKF) with both adaptivity and robustness for AUV navigation. However, these approaches are based on a loosely integrated navigation system and do not make effective use of the beam of DVL. Moreover, the SINS/DVL navigation system has to face instantaneous outliers and gradually changing outliers. Therefore, these models fail to estimate the state vector where the observation noise increases and thus affects the robustness of the navigation system.

To address the above issue, in this paper, a novel tightly integrated navigation model is established for SINS, DVL, and PS based on the effects of DVL water-track and bottom-track velocity measurement models. Furthermore, we present a robust IMM model (RIMM), which is based on a DVL beam processing strategy. This strategy includes data anomaly detection and the virtual beam (VB) method by a tightly integrated system and a modified Markov transfer probability matrix. RIMM ensures that each model can be converted quickly into outliers and outlier noise.

The rest of this paper is organized as follows. Section 2 introduces the SINS, DVL, and PS systems and establishes the SINS, DVL, and PS tightly integrated navigation model based on the DVL water-track and bottom-track velocity measurement models. Section 3 explains the principle of the proposed RIMM algorithm. Section 4 verifies the proposed model and algorithm by comparing simulations with the existing methods. The paper is ended by providing conclusions in Section 5.

## 2. Materials and Methods

**Figure 1:**
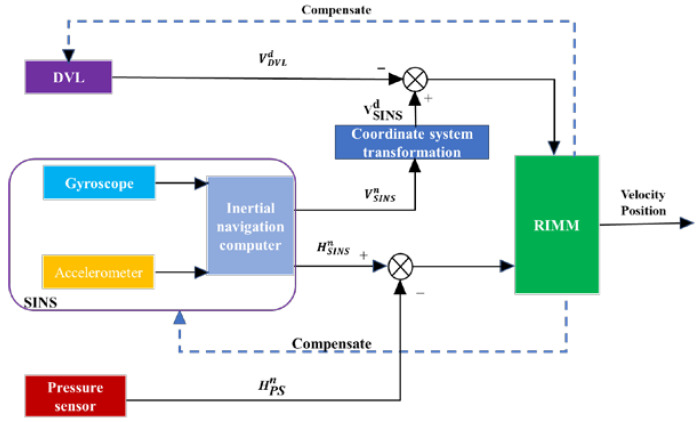
Tightly coupled navigation system of SINS, DVL, and PS.

An AUV navigation system is composed of SINS, DVL, and a pressure sensor (PS), as shown in Figure 1. SINS provides required velocity, attitude, and position information through a gyroscope and an accelerometer. DVL has a four-beam Janus structure, and it does not require external information to reduce the error accumulation in SINS. The PS is a device that provides depth information for AUV, and it is usually considered an alternative to DVL. To make full use of the effective information from the sensor, the system adopts a tightly coupled navigation structure.

In Figure 1, n  represents the navigation frame with an east–north–up (ENU) orientation, d  represents the DVL body frame aligned with a right–forward–up orientation, and beam represents the original four-channel body frame.

### 2.1. DVL Working Model

**Figure 2:**
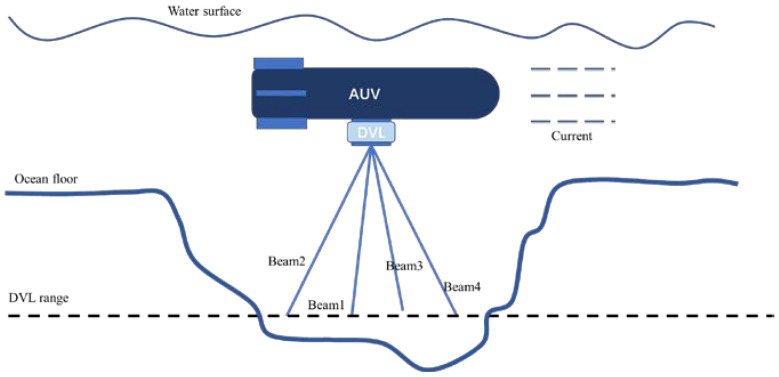
DVL failure condition.

Bottom-track and water-track velocity measurements are the two main modes of operation for DVL on AUVs. The bottom-track measurement mode is usually used in the model of the SINS and DVL integrated navigation system for AUVs. Nevertheless, as shown in Figure 2, in practice, the application of this mode can be affected by the irregular ocean floor and changes in the angle between the AUV and the ocean floor, where DVL cannot offer its bottom-track velocity measurement continuously. In such cases, the water-track velocity measurement mode needs to be applied. For this reason, DVL needs to establish a water-track velocity measurement model.

The current velocity changes over time and is influenced by the wind speed, temperature, salinity, and topography of the environment. The current velocity is a slowly changing process. Therefore, to reflect the changing characteristics of the current velocity, a first-order Markov process is used to simulate the process of water velocity changes [33,34].

Suppose that the correlation distance in the area is $L_{\mathrm{auv}}={[L}_{E}{,L}_{N}{,L}_{U}]$ and the velocity of the vehicle is $V_{\mathrm{auv}}=[V_{E}{,V}_{N}{,V}_{U}]$. The correlation time of the current velocity is:

(1) $${\left[\begin{array}{ccc} \tau_{E} & \tau_{N} & \tau_{U} \end{array}\right]}^{T}=[\begin{array}{ccc} \frac{L_{E}}{V_{E}} & \frac{L_{N}}{V_{N}} & \frac{L_{U}}{V_{U}} \end{array}{]}^{T}$$

where $\;\tau_{E}$, $\tau_{N}$, and $\tau_{U}$ are the correlation time in the three directions; $L_{E}$, $L_{N}$, and $L_{U}$ are the correlation distances eastward, northward, and upward, respectively, that remain constant within a certain area; and $V_{E}$, $V_{N}$, and $V_{U}$ represent the eastward, northward, and upward velocities, respectively, in frame n. The velocity of the vehicle changes with time, and thus different velocities match various correlation times. The higher the speed of the AUV, the lower the correlation time.

Ignoring errors, the first-order Markov process is used to describe the change in the water current velocity:

(2) $${\left[\begin{array}{ccc} {\dot{V}}_{\mathrm{CE}}^{n} & {\dot{V}}_{\mathrm{CN}}^{n} & {\dot{V}}_{\mathrm{CU}}^{n} \end{array}\right]}^{T}=\mathrm{diag}(\begin{array}{ccc} \tau_{E} & \tau_{N} & \tau_{U} \end{array})[\begin{array}{ccc} V_{\mathrm{CE}}^{n} & V_{\mathrm{CN}}^{n} & V_{\mathrm{CU}}^{n} \end{array}{]}^{T}$$

where $V_{\mathrm{CE}}^{n}$,${\;V}_{\mathrm{CN}}^{n}$, and $V_{\mathrm{CU}}^{n}$ are the current velocities in the east, north, and upward directions in frame n, respectively.

**Figure 3:**
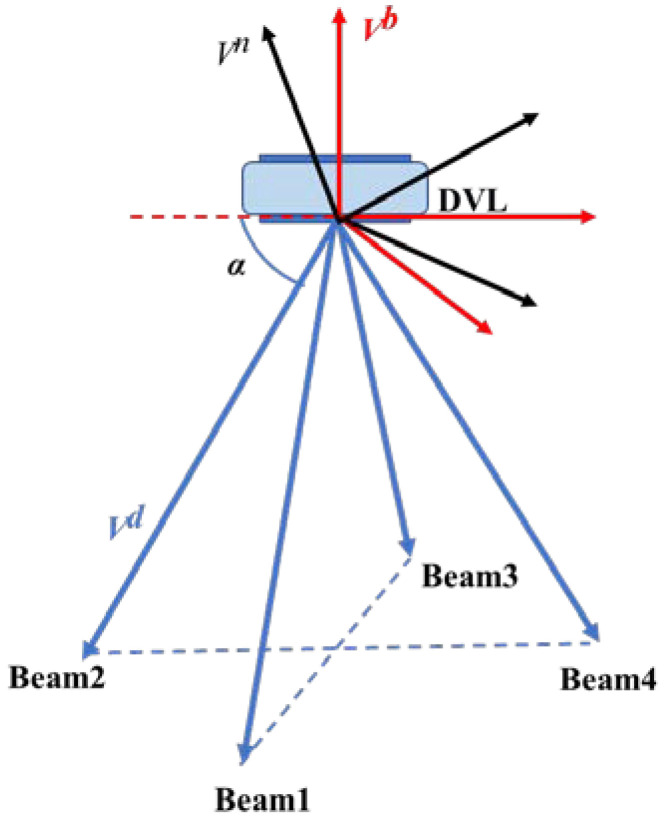
Relationships between different frames.

For DVL with the four-beam Janus structure, the current velocity affects the velocity of DVL beams. To assess the effect of the current velocity on the effective information of each DVL beam, it is necessary to convert the current velocity in frame n to frame d. The conversion relationships are as follows:

(3) $$V_{C}^{d}=C_{b}^{d}C_{n}^{b}V_{C}^{n}$$

where $V_{C}^{n}=[\begin{array}{ccc} V_{\mathrm{CE}}^{n} & V_{\mathrm{CN}}^{n} & V_{\mathrm{CU}}^{n} \end{array}{]}^{T}$ and  b  is the AUV body frame. $C_{n}^{b}$ is the direction cosine matrix of transformation from frame n to frame b, and $C_{b}^{d}$ is the direction cosine matrix of transformation from frame b to d frame. The relationships between different frames are shown in Figure 3, where the frame is represented by a red line and $V^{b}$ indicates the velocity in frame b. Frame n is also represented by a black line, and $V^{n}$ indicates the velocity in the n frame. The blue line is drawn to represent the DVL frame (frame d), and $C_{b}^{d}$ can be expressed as:

(4) $$C_{b}^{d}=\left[\begin{array}{ccc} 0 & \cos\alpha & -\sin\alpha \\ \cos\alpha & 0 & -\sin\alpha \\ 0 & -\cos\alpha & -\sin\alpha \\ -\cos\alpha & 0 & -\sin\alpha \end{array}\right]$$

where α represents the horizontal angle between the beams and the AUV. Usually, ${\alpha =70}^{{}^{\circ}}$.

Ignoring sensor errors, the velocity of DVL under frame d is defined as:

(5) $$V_{DVL}^{d}={\left[\begin{array}{cccc} V_{DVL\_1}^{d} & V_{DVL\_2}^{d} & V_{DVL\_3}^{d} & V_{DVL\_4}^{d} \end{array}\right]}^{T}$$

where $V_{DVL\_1}^{d}$,${\;V}_{DVL\_2}^{d}$,${\;V}_{DVL\_3}^{d}$, and $V_{DVL\_4}^{d}$ represent the true velocity information for each beam of DVL in frame d.

The DVL water-track measurement can be modeled as:

(6) $${\tilde{V}}_{DVL\_C}^{d}=V_{DVL}^{d}\left(1+\delta K\right)+V_{C}^{d}+\omega_{c}$$

where δK is the scale factor error of DVL and $\omega_{c}$ represents the white noise. The DVL bottom-track measurement can also be modeled as [14]:

(7) $${\tilde{V}}_{DVL\_B}^{d}=V_{DVL}^{d}\left(1+\delta K\right)+V_{B}^{d}+\omega_{b}$$

where $V_{B}^{d}=\left[\begin{array}{cccc} b_{1} & b_{2} & b_{3} & b_{4} \end{array}\right]$ represents the biases of the four beams of DVL and $\omega_{b}$ represents the white noise.

### 2.2. SINS, DVL, and PS Tightly Coupled Integrated Method

In contrast to the traditional SINS, DVL, and PS tightly integrated navigation methods, the tightly integrated navigation method used in this paper is based on the velocity variation of the DVL beam, applying the DVL bottom-track model and the water-track model. The state equation of the tightly coupled integrated method under frame n can be expressed as:

(8) $$\dot{X}=FX+GW$$

where X is the state vector, F represents the state transition matrix, G is the system noise matrix, and W denotes the process noise vector. The state vector X is 24-dimensional and can be expressed as follows:

(9) $$X=[\emptyset_{x}\emptyset_{y}\emptyset_{z}{\;\delta V}_{E}^{n}{\;\delta V}_{N}^{n}{\;\delta V}_{U}^{n}\;\delta \lambda \;\delta L\;\delta h\;\nabla_{x}\nabla_{y}\nabla_{z}{\;\varepsilon }_{x}{\;\varepsilon }_{y}{\;\varepsilon }_{z}\;b_{1}{\;b}_{2}{\;b}_{3}{\;b}_{4}{\;\delta K\;V}_{\mathrm{CE}}^{n}{\;V}_{\mathrm{CN}}^{n}{\;V}_{\mathrm{CU}}^{n}{\;b}_{PS}{]}^{T}$$

where $\emptyset_{x}$, $\emptyset_{y}$, and $\;\emptyset_{z}$ denote SINS misalignment angles; ${\delta V}_{E}^{n},{\;\delta V}_{N}^{n}$, and ${\delta V}_{U}^{n}\;$ are SINS velocity errors; δλ, δL, and *δh* denote the longitude, latitude, and height error of SINS, respectively; $\nabla_{x}$, $\nabla_{y}$, and $\nabla_{z}$ denote accelerometer biases of SINS; $\varepsilon_{x}$, $\varepsilon_{y}$, and $\varepsilon_{z}$ denote gyroscope biases in three directions of frame b; and $b_{PS}$ is the PS bias. The system state transition matrix F can be written as follows:

(10) $$F=\left[\begin{array}{cc} \begin{array}{ccccc} F_{11} & F_{12} & F_{13} & {-C}_{b}^{n} & 0_{3\times 3}\\ F_{21} & F_{22} & F_{23} & 0_{3\times 3} & C_{b}^{n}\\ 0_{3\times 3} & F_{32} & F_{33} & C_{b}^{n} & 0_{3\times 3} \end{array} & 0_{9\times 9}\\ 0_{15\times 15} & \end{array}\right]$$

where $F_{11}$, $F_{12}$, $F_{13}$,${\;F}_{21}$,${\;F}_{22}$,${\;F}_{23}$,${\;F}_{32}$, and $F_{33}$ are defined as follows:

$$F_{11}=\left[\begin{array}{ccc} 0 & w_{\mathrm{ie}}\sin L+\frac{V_{E}\tan L}{R_{N}+h} & -w_{\mathrm{ie}}\cos L-\frac{V_{E}\tan L}{R_{N}+h}\\ {-w}_{\mathrm{ie}}\sin L+\frac{V_{E}\tan L}{R_{N}+h} & 0 & \frac{V_{N}}{R_{M}+h}\\ w_{\mathrm{ie}}\cos L+\frac{V_{E}\tan L}{R_{N}+h} & \frac{V_{E}}{R_{M}+h} & 0 \end{array}\right]$$

$$F_{12}=\left[\begin{array}{ccc} 0 & \frac{1}{R_{M}+h} & 0\\ \frac{1}{R_{N}+h} & 0 & 0\\ \frac{\tan L}{R_{N}+h} & 0 & 0 \end{array}\right]$$

$$F_{13}=\left[\begin{array}{ccc} 0 & 0 & \frac{V_{N}}{{{(R}_{M}+h)}^{2}}\\ {-w}_{\mathrm{ie}}\sin L & 0 & -\frac{V_{E}}{{{(R}_{N}+h)}^{2}}\\ w_{\mathrm{ie}}\cos L+\frac{V_{E}\sec^{2}L}{R_{N}} & 0 & -\frac{V_{E}\tan L}{{{(R}_{N}+h)}^{2}} \end{array}\right]$$

$$F_{21}=\left[\begin{array}{ccc} 0 & -f_{U}^{n} & f_{N}^{n}\\ f_{U}^{n} & 0 & -f_{E}^{n}\\ -f_{N}^{n} & f_{E}^{n} & 0 \end{array}\right]$$

$$F_{22}=\left[\begin{array}{ccc} \frac{V_{E}\tan L-V_{U}}{R_{N}+h} & {2w}_{\mathrm{ie}}\sin L+\frac{V_{E}\tan L}{R_{N}+h} & {-2w}_{\mathrm{ie}}\cos L-\frac{V_{E}\tan L}{R_{N}+h}\\ {-2w}_{\mathrm{ie}}\sin L-\frac{2V_{E}\tan L}{R_{N}+h} & -\frac{V_{U}}{R_{M}+h} & -\frac{V_{N}}{R_{M}+h}\\ {-2w}_{\mathrm{ie}}\cos L+\frac{2V_{E}}{R_{N}+h} & \frac{2V_{N}}{R_{M}+h} & 0 \end{array}\right]$$

$$F_{23}={(V}^{n}\times )\left(\left[\begin{array}{ccc} 0 & 0 & \frac{V_{N}}{{{(R}_{M}+h)}^{2}}\\ {-2w}_{\mathrm{ie}}\sin L & 0 & -\frac{V_{E}}{{{(R}_{N}+h)}^{2}}\\ {-2w}_{\mathrm{ie}}\cos L+\frac{V_{E}\sec^{2}L}{R_{N}} & 0 & \frac{V_{E}\tan L}{{{(R}_{N}+h)}^{2}} \end{array}\right]\right)$$

$$F_{32}=\left[\begin{array}{ccc} 0 & \frac{1}{R_{M}+h} & 0\\ \frac{\sec L}{R_{N}+h} & 0 & 0\\ 0 & 0 & 1 \end{array}\right]$$

$$F_{33}=\left[\begin{array}{ccc} 0 & 0 & -\frac{V_{N}}{{{(R}_{M}+h)}^{2}}\\ \frac{V_{E}\tan L\sec L}{R_{N}+h} & 0 & -\frac{V_{E}\sec L}{{{(R}_{N}+h)}^{2}}\\ 0 & 0 & 0 \end{array}\right]$$

where $R_{M}\;$ and $R_{N}$ are the transverse radius and meridian radius of the Earth, respectively, L is the latitude, h denotes the height, and $w_{\mathrm{ie}}$ is the Earth’s rotation rate. The matrices G and W are:

(11) $$G=\left[\begin{array}{cc} I_{3\times 3} & 0_{3\times 3}\\ 0_{3\times 3} & I_{3\times 3}\\ 0_{18\times 3} & 0_{18\times 3} \end{array}\right],\;W=\left[\begin{array}{c} w_{g}^{n}\\ w_{a}^{n} \end{array}\right]$$

The measurement equation is made up of three components, including the difference between the SINS’ velocity and the DVL bottom-track velocity, the difference between the SINS’ velocity and the DVL water-track velocity, and the difference between the SINS’ height and PS measurement value.

Ignoring sensor errors, the velocity of SINS in different coordinate systems is defined as follows:

(12) $$\left\{\begin{array}{l} V_{SINS}^{b}={\left[\begin{array}{ccc} V_{x}^{b} & V_{y}^{b} & V_{z}^{b} \end{array}\right]}^{T}\\ V_{SINS}^{n}={\left[\begin{array}{ccc} V_{SINS\_N}^{n} & V_{SINS\_E}^{n} & V_{SINS\_U}^{n} \end{array}\right]}^{T}\\ V_{SINS}^{d}={\left[\begin{array}{cccc} V_{SINS\_1}^{d} & V_{SINS\_2}^{d} & V_{SINS\_3}^{d} & V_{SINS\_4}^{d} \end{array}\right]}^{T} \end{array}\right.$$

where $V_{SINS}^{b}$ denotes the velocity of SINS under the b frame, $V_{SINS}^{b}$ denotes the velocity of SINS under the n frame, and $V_{SINS}^{d}$ denotes the velocity of SINS under the d frame. The relationship between the velocity of SINS and the velocity of DVL is:

(13) $$V_{DVL}^{d}=V_{SINS}^{d}=C_{b}^{d}C_{n}^{b}V_{SINS}^{n}$$

Considering the installation angle error between the gyroscope and DVL, it is assumed that the installation angle’s error after calibration compensation is:

(14) $$\varphi =[\begin{array}{ccc} \varphi_{x} & \varphi_{y} & \varphi_{z} \end{array}]$$

Furthermore, $C_{b}^{d}$, considering the installation angle error between the gyroscope and DVL, is expressed as:

(15) $$\left\{\begin{array}{c} {\tilde{C}}_{b}^{d}=C_{b}^{d}\left(I_{3\times 3}+\varphi \times \right)\\ {\tilde{C}}_{n}^{b}=C_{n}^{b}\left(I_{3\times 3}+\emptyset \times \right) \end{array}\right.$$

where $\emptyset =[\emptyset_{x}\;\emptyset_{y}\;\emptyset_{z}]$.

The velocity information of SINS can be expressed in the d frame as:

(16) $${\tilde{V}}_{SINS}^{d}={\tilde{C}}_{b}^{d}{\tilde{C}}_{n}^{b}\left(V_{SINS}^{n}+{\delta V}_{SINS}^{n}\right)\approx {\tilde{C}}_{b}^{d}C_{n}^{b}V_{SINS}^{n}+{\tilde{C}}_{b}^{d}C_{n}^{b}{\delta V}_{SINS}^{n}+{\tilde{C}}_{b}^{d}C_{n}^{b}\emptyset \times V_{SINS}^{n}$$

According to Equations (13) and (16):

(17) $${\tilde{V}}_{SINS}^{d}=V_{DVL}^{d}+{\tilde{C}}_{b}^{d}C_{n}^{b}{\delta V}_{SINS}^{n}{-\tilde{C}}_{b}^{d}C_{n}^{b}V_{SINS}^{n}\times \emptyset$$

The observation equation of the integrated navigation system contains three parts. The observation equation for the tightly integrated navigation system of the SINS velocity and DVL water-track velocity is:

(18) $$Z_{1}={\tilde{V}}_{DVL\_C}^{d}-{\tilde{V}}_{SINS}^{d}={\delta KV}_{DVL}^{d}+V_{C}^{d}+\omega_{c}-{\tilde{C}}_{b}^{d}C_{n}^{b}{\delta V}_{SINS}^{n}+{\tilde{C}}_{b}^{d}C_{n}^{b}V_{SINS}^{n}\times \emptyset$$

The difference between the SINS velocity and DVL bottom-track velocity is taken as the observation:

(19) $$Z_{2}={\tilde{V}}_{DVL\_B}^{d}-{\tilde{V}}_{SINS}^{d}={\delta KV}_{DVL}^{d}+V_{B}^{d}+\omega_{b}-{\tilde{C}}_{b}^{d}C_{n}^{b}{\delta V}_{SINS}^{n}+{\tilde{C}}_{b}^{d}C_{n}^{b}V_{SINS}^{n}\times \emptyset$$

and the PS error measurement model is:

(20) $${\tilde{H}}_{PS}=H_{PS}+{\delta b}_{PS}+w_{PS}$$

where ${\tilde{H}}_{PS}$ represents the sensor measurement, $H_{PS}$ represents the true value, ${\delta b}_{PS}$ represents the PS biases, and $w_{PS}$ represents the white noise.

The difference between the SINS height and the PS measurement is therefore taken as the observation:

(21) $$Z_{3}={\tilde{H}}_{PS}-{\tilde{H}}_{SINS}=H_{PS}+{\delta b}_{PS}+w_{PS}-H_{SINS}-{\delta h=\delta b}_{PS}+w_{PS}-\delta h$$

where ${\tilde{H}}_{SINS}$ represents the SINS height measurement and $H_{SINS}$ represents the SINS true height value.

Finally, the system observation equation is expressed as:

(22) $$Z=\left[\begin{array}{c} Z_{1}\\ Z_{2}\\ Z_{3} \end{array}\right]=HX+V$$

where the transfer matrix H is:

(23) $$H=\left[\begin{array}{c} \begin{array}{cccccc} H_{C1} & H_{C2} & 0_{4\times 13} & V_{DVL}^{d} & C_{b}^{d}C_{n}^{b} & 0 \end{array}\\ \begin{array}{cccccc} H_{B1} & H_{B2} & 0_{4\times 9} & I_{4\times 4} & V_{DVL}^{d} & 0_{4\times 4} \end{array}\\ \begin{array}{cccc} 0_{1\times 8} & -1 & 0_{1\times 14} & 1 \end{array} \end{array}\right]$$

(24) $$\left\{\begin{array}{l} H_{C1}={\tilde{C}}_{b}^{d}C_{n}^{b}V_{SINS}^{n}\times \\ H_{C2}=-{\tilde{C}}_{b}^{d}C_{n}^{b}\\ H_{B1}={\tilde{C}}_{b}^{d}C_{n}^{b}V_{SINS}^{n}\times \\ H_{B2}={-\tilde{C}}_{b}^{d}C_{n}^{b} \end{array}\right.$$

and the measurement noise vector V is:

(25) $$V={\left[\begin{array}{ccc} \omega_{c} & \omega_{b} & w_{PS} \end{array}\right]}^{T}$$

## 3. Robust Interacting Multiple Models

**Figure 4:**
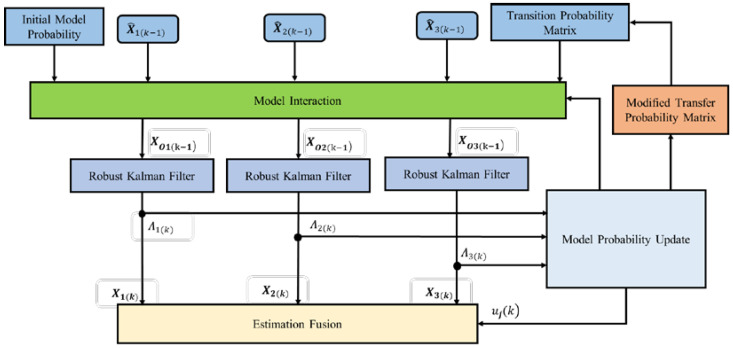
Robust IMM diagram.

To improve the stability of SINS, DVL, and PS integrated navigation systems, we propose the RIMM. The RIMM system includes three models: SINS, DVL, and PS tightly integrated navigation model without DVL beam error, SINS/DVL tightly integrated navigation model with water-track velocity error mode, and SINS, DVL, and PS tightly integrated navigation model with bottom-track velocity error mode. The SINS, DVL, and PS tightly integrated navigation model without DVL beam error is the main model of the system. The process of the RIMM algorithm includes five steps, including model interaction, model filtering, model probability update, modified transfer probability matrix, and estimation fusion (Figure 4).

### 3.1. Model Interaction

By initializing the model conditions, we obtain the state vector and covariance matrix of each robust Kalman filter input at the current moment of the model. The parameters are calculated as follows.

The predicted model probability from model *i* to model *j* is:

(26) $$u_{\mathrm{ij}}\left(k-1\right){=p}_{\mathrm{ij}}u_{i}(k-1)/\overset{r}{\underset{i=1}{\sum }}p_{\mathrm{ij}}u_{i}\left(k-1\right)$$

where $p_{\mathrm{ij}}$ is a Markov transition probability matrix representing the probability of conversion from model *i* to model *j*, $u_{i}\left(k-1\right)$ is the probability of model i at epoch k−1, and r  is the number of models. Here, we set r=3.

The mixing state vector and its estimated covariance of each filter are updated according to the predicted model probability:

(27) $${\hat{X}}_{\mathrm{Oj}(k-1)}=\overset{r}{\underset{i=1}{\sum }}{\hat{X}}_{i\left(k-1\right)}u_{\mathrm{ij}}\left(k-1\right)$$

(28) $$P_{\mathrm{Oj}(k-1)}=\overset{r}{\underset{i=1}{\sum }}{(P}_{i\left(k-1\right)}+[{\hat{X}}_{i\left(k-1\right)}-{\hat{X}}_{\mathrm{Oj}\left(k-1\right)}]{\left[{\hat{X}}_{i\left(k-1\right)}-{\hat{X}}_{\mathrm{Oj}\left(k-1\right)}\right]}^{T}{)u}_{\mathrm{ij}}\left(k-1\right)$$

where ${\hat{X}}_{i\left(k-1\right)}$ and $P_{i\left(k-1\right)}$ are the state estimate and its covariance matrix of filter i at the last epoch, respectively, ${\hat{X}}_{\mathrm{Oj}(k-1)}$ is the mixing state vector estimate of filter j in the current epoch, and $P_{\mathrm{Oj}(k-1)}$ is its corresponding covariance matrix.

### 3.2. Robust Kalman Filter

To improve the stability of the system, a robust Kalman filter algorithm is proposed to avoid the influence of DVL beam errors on navigation stability. The state equation and observation equation are shown in Equations (8) and (22), respectively. Here, we transform Equations (8) and (22) into the discrete-time formula:

(29) $$\left\{\begin{array}{l} X_{j\left(k\right)}=\emptyset_{k,k-1}X_{j\left(k-1\right)}+G_{k}W_{k}\\ Z_{k}=H_{k}X_{j\left(k\right)}+V_{j(k)} \end{array}\right.$$

The update and prediction processes of the Kalman filter are [35]:

(30) $${\hat{X}}_{j(k,k-1)}=\emptyset_{k,k-1}{\hat{X}}_{\mathrm{Oj}(k,k-1)}$$

where ${\hat{X}}_{j(k,k-1)}$ is the predicted state estimate of filter *j* and $\emptyset_{k,k-1}$ is the state transition matrix.

(31) $$P_{j(k,k-1)}=\emptyset_{k,k-1}P_{0j(k-1)}\emptyset_{k,k-1}^{T}+G_{k}Q_{k}G_{k}^{T}$$

where $P_{j(k,k-1)}$ and $P_{0j(k-1)}$ are the predicted estimate covariance and updated estimate covariance, respectively, and $Q_{k}$ is the state variance matrix, which remains the same for all the filters.

(32) $$r_{j(k)}=(Z_{k}-H_{k}{\hat{X}}_{j\left(k,k-1\right)})$$

where $r_{j\left(k\right)}$ is the residual of Kalman.

(33) $$K_{j(k)}=P_{j\left(k,k-1\right)}H_{k}^{T}{{(H}_{k}P_{j\left(k,k-1\right)}H_{k}^{T}+R_{j(k)})}^{-1}$$

(34) $$X_{j(k)}={\hat{X}}_{j\left(k,k-1\right)}+K_{j(k)}r_{j(k)}$$

(35) $$P_{j(k)}=({I-K}_{j\left(k\right)}H_{k})P_{j(k,k-1)}$$

$K_{j(k)}$ is the Kalman gain, $R_{j(k)}$ is the observation variance matrix of different filters, and $P_{j\left(k\right)}$ is the updated estimate covariance.

In the Kalman filter process, outliers are found by monitoring large-beam measurement errors [14]. The velocity differences between SINS and DVL are then calculated under the d frame:

(36) $${\tilde{V}}_{DVL}^{d}-{\tilde{V}}_{SINS}^{d}=V_{\mathrm{error}}^{d}$$

Normally, $V_{\mathrm{error}}^{d}$ follows a zero-mean Gaussian distribution; nevertheless, in cases where the DVL beam is affected by the external environment, $V_{error\;}^{d}$ does not follow the zero-mean Gaussian distribution. Therefore, threshold *β* can be used to determine whether the beam measurement is available.

(37) $$\mathrm{Normal}:\;V_{\mathrm{error}}^{d}<\beta \;\mathrm{Abnormal}:\;V_{\mathrm{error}}^{d}\geq \beta$$

In [21], a DVL beam processing strategy is introduced, which includes data anomaly detection and the virtual beam (VB) method. As shown in Figure 3, the method assumes that the DVL beam has the following characteristics:

(38) $$V_{DVL\_1}^{d}{=-V}_{DVL\_3}^{d}\;V_{DVL\_2}^{d}{=-V}_{DVL\_4}^{d}$$

If VB is not available, the beam measurement is isolated, and only the SINS and PS integrated navigation is used.

### 3.3. Model Probability Update

Based on the Gaussian assumption, the likelihood function of each model is:

(39) $$f_{j}\left(k\right)=\exp\left(-\frac{1}{2}r_{j\left(k\right)}^{T}A_{j\left(k\right)}^{-1}r_{j\left(k\right)}\right)/{\left({\left(2\pi \right)}^{m}\left|A_{j\left(k\right)}\right|\right)}^{1/2}$$

where m is the dimension of the observation vector. The model probability is updated as:

(40) $$\Lambda_{j(k)}=\overset{r}{\underset{i=1}{\sum }}p_{\mathrm{ij}}u_{i}\left(k-1\right)$$

(41) $$u_{j}{(k)=f}_{j}{(k)\Lambda }_{j}/\overset{r}{\underset{j=1}{\sum }}f_{j}\left(k\right)\Lambda_{j}$$

### 3.4. Modified Transfer Probability Matrix

The traditional IMM algorithm artificially selects a fixed transition probability matrix according to prior knowledge. However, the motion environment of the AUV is uncertain, and a fixed transition probability matrix inevitably influences the sensors’ data fusion. Generally, when the motion state of the AUV changes, the probability of changing the mismatched model to a matched model increases, while the probability of maintaining the existing model decreases. To ensure that the transfer probability matrix of the IMM algorithm is changed according to the motion state of the AUV, the correction parameter is defined as [36]:

(42) $$\tau_{ij}=\frac{p_{k}^{ji}\cdot \Lambda_{j(k)}}{p_{k}^{ij}\cdot \Lambda_{j(k)}}$$

For i≠j,

(43) $$p_{ij}^{\prime }={\left(\tau_{ij}\right)}^{\alpha }p_{ij}$$

where $p_{ij}^{\prime }$ is the modified transfer probability matrix and α is the modified factor. The larger the value of α, the faster the correction. The elements on the main diagonal are

(44) $$p_{ii}^{\prime }{=1-(\tau }_{i1}{)}^{\alpha }p_{i1}-\cdots {-(\tau }_{\mathrm{ir}}{)}^{\alpha }p_{\mathrm{ir}}$$

Given the principle of the dominance of the main diagonal, we set a threshold σ. If after probability correction the value of the principal diagonal element is less than σ, the correction is made according to the methods of Equations (45) and (46).

(45) $$p_{ii}^{\prime }=\sigma$$

(46) $$p_{ij}^{\prime }=\left(1-\sigma \right)\frac{p_{ij}}{{1-p}_{ii}},\;i\neq j$$

### 3.5. Estimation Fusion

Based on the model probability update in Section 3.3, the state vector and its covariance matrix of each filter are fused to calculate the combined state estimation and covariance matrix as the final filtering output:

(47) $${\hat{X}}_{k}=\overset{r}{\underset{j=1}{\sum }}{\hat{X}}_{j(k)}u_{j}\left(k\right)$$

(48) $$P_{k}=\overset{r}{\underset{j=1}{\sum }}u_{j}\left(k\right){[P}_{j\left(k\right)}{+(\hat{X}}_{j\left(k\right)}{-\hat{X}}_{k}{)(\hat{X}}_{j\left(k\right)}{-\hat{X}}_{k}{)}^{T}]$$

## 4. Results and Discussion

In this section, simulations are used to evaluate the feasibility of the proposed algorithm. Firstly, the RIMM algorithm is compared with the traditional loosely integrated navigation algorithm and the tightly integrated navigation algorithm, assuming that DVL is working properly. Then, in the case of abnormal DVL operation, the RIMM algorithm is compared with the traditional loosely integrated navigation algorithm and the tightly integrated navigation algorithm. Finally, the RIMM algorithm and IMM algorithm are compared and analyzed for different conditions of DVL.

In addition, to compare the performance differences between the three methods, root-mean-squared error (RMSE), mean error, and maximum deviation are used to describe the statistical properties. The RMSE is the arithmetic square root of the variance that reflects the degree of dispersion of a data set. The smaller the RMSE value, the more accurate the prediction model is in describing the experimental data. RMSE is defined as:

(49) $$\mathrm{RMSE}=\sqrt{\frac{\sum_{i=1}^{N}{\left(x_{i}-\bar{x}\right)}^{2}}{N}}$$

**Figure 5:**
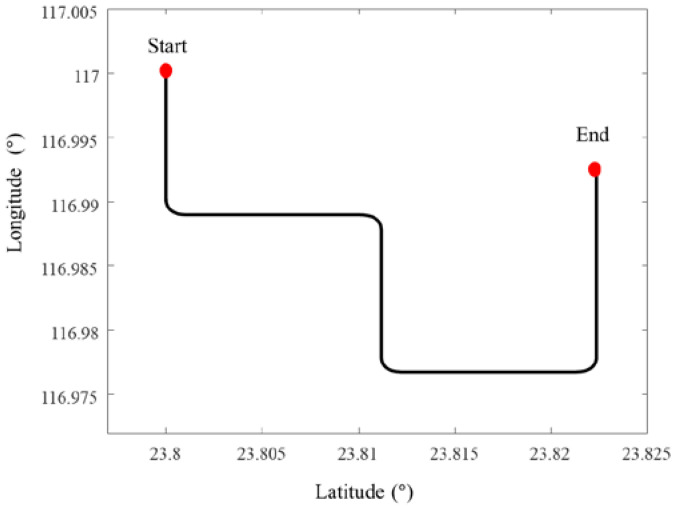
Motion trajectory of AUV.

**Figure 6:**
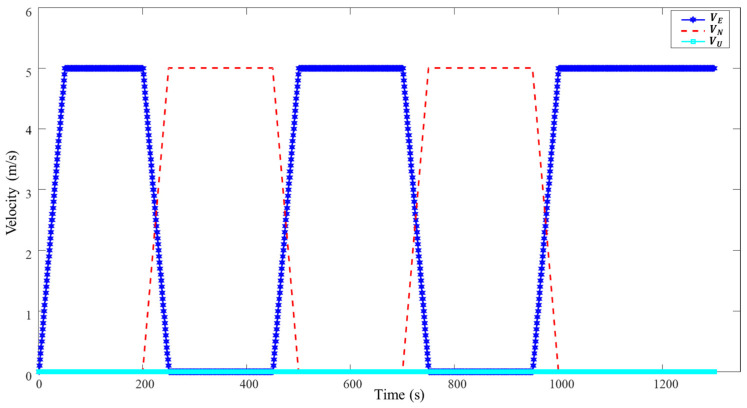
Velocity variation of AUV.

The whole simulation time lasts for 1300 s, which includes acceleration, deceleration, uniform, and turning motion, as shown in Figure 5 and Figure 6. The AUV starts at [$23.8^{{}^{\circ}}\;N,117^{{}^{\circ}}\;E$] at a depth of 5 m. The sampling frequencies for IMU, DVL, and PS are 200 Hz, 1 Hz, and 1 Hz, respectively. The main performance parameters of IMU, DVL, and PS are listed in Table 1. Because of the different sampling frequencies at each sensor, synchronization of the sensor before data fusion is required. In this paper, the least-squares registration method is used for time synchronization [37].

In the simulation process, the initial attitude is ${\theta =0}^{{}^{\circ}}{,\;\gamma =0}^{{}^{\circ}}{,\;\psi =0}^{{}^{\circ}}$, and the initial velocity is 0 m/s. In the SINS/DVL/PS integrated navigation system, the installation error between SINS and DVL is [$0.001^{{}^{\circ}},0.002^{{}^{\circ}},0.007^{{}^{\circ}}$], and the scale factor of DVL is 0.002. The AUV simulates at medium sea level; the attitude angles of the AUV are

(50) $$\left\{\begin{array}{c} {\theta =\theta }_{m}\sin(\frac{2\pi }{5}{t+\theta }_{0})\\ {\gamma =\gamma }_{m}\sin(\frac{2\pi }{7}{t+\gamma }_{0})\\ {\psi =\psi }_{m}\sin(\frac{2\pi }{6}{t+\psi }_{0}) \end{array}\right.$$

where $\theta_{m}=3^{{}^{\circ}}$, $\gamma_{m}=4^{{}^{\circ}}$, and $\psi_{m}=3^{{}^{\circ}}$, and the rolling periods are 5, 7, and 6 s. The initial phases are $\theta_{0}=0^{{}^{\circ}},\gamma_{0}=0^{{}^{\circ}},$. and $\;\psi_{0}=0^{{}^{\circ}}$.

### 4.1. DVL Working Properly

**Figure 7:**
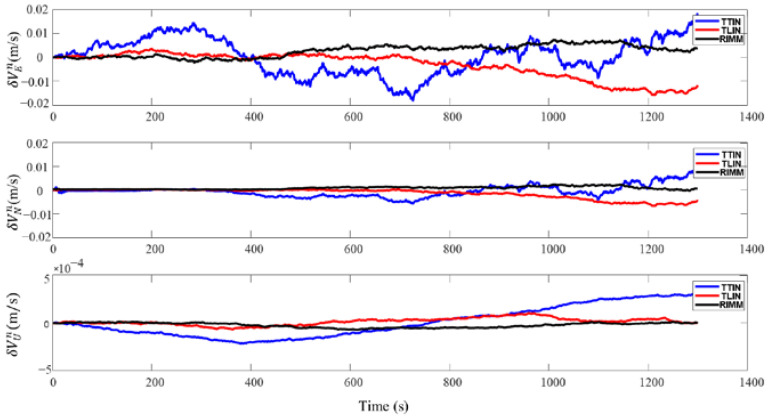
Velocity errors of three algorithms when DVL working properly.

In this section, DVL is in a stable working state. In Figure 7, the loosely integrated navigation algorithm (TLIN) is shown by a red line, the tightly integrated navigation (TTIN) is depicted by a blue line, and the RIMM algorithm is illustrated by a black line. Figure 7 shows that the velocity errors of the three methods are stable in the three-dimensional direction. Table 2 also shows that there is no significant difference between them. The maximum deviations of TTIN, TLIN, and RIMM in the ${\delta V}_{E}^{n}$ are 0.0182 m/s, −0.0157 m/s, and 0.0074 m/s, respectively, and the maximum deviations of TTIN, TLIN, and RIMM in the ${\delta V}_{N}^{n}$ are 0.0099 m/s, −0.0066 m/s, and 0.0024 m/s, respectively.

**Figure 8:**
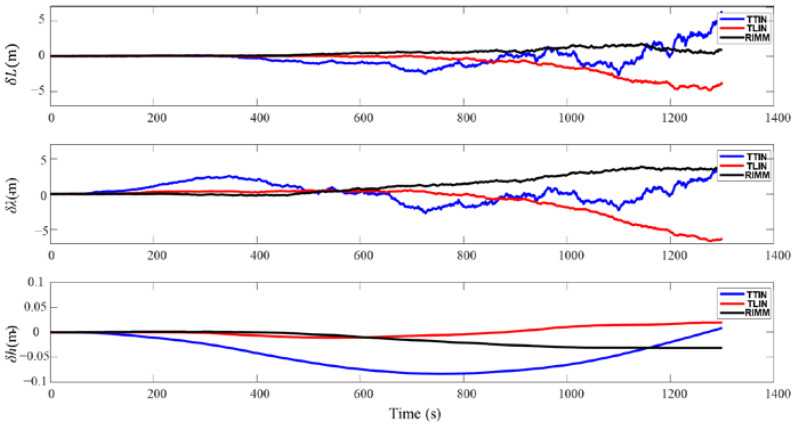
Position errors of three algorithms when DVL working properly.

In terms of position error, the PS can provide high-precision depth information during the motion of the AUV. Figure 8 shows that the height position always maintains a stable high precision throughout the navigation process. Table 3 also shows that the maximum height deviation position has a −0.084 m error during the entire simulation process. Furthermore, the velocity error in the $\;\delta V_{U}^{n}$ also remains stable because of the PS, and the maximum velocity errors of TTIN, TLIN, and RIMM are 0.00030 m/s, 0.00010 m/s, and 0.00007 m/s, respectively. Therefore, when DVL is in a stable working state, the three integrated navigation algorithms show stable navigation and positioning accuracy.

### 4.2. DVL Works in a Complex Environment

The navigation accuracy of DVL is also affected by the external operating conditions of the AUV. We consider six failure cases for the DVL beam, as shown in Table 4, where DVL is in the bottom-track measurement mode in a complex environment. In the tightly integrated navigation mode, the integrated navigation operation modes corresponding to different DVL beam failures are SINS, DVL, and PS and SINS and PS. In cases 4, 5, and 6, only SINS and PS can be used for navigation. To compare the proposed RIMM with the loosely integrated navigation and the tightly integrated navigation, the main considerations in this section are cases 1, 2, and 3.

**Figure 9:**
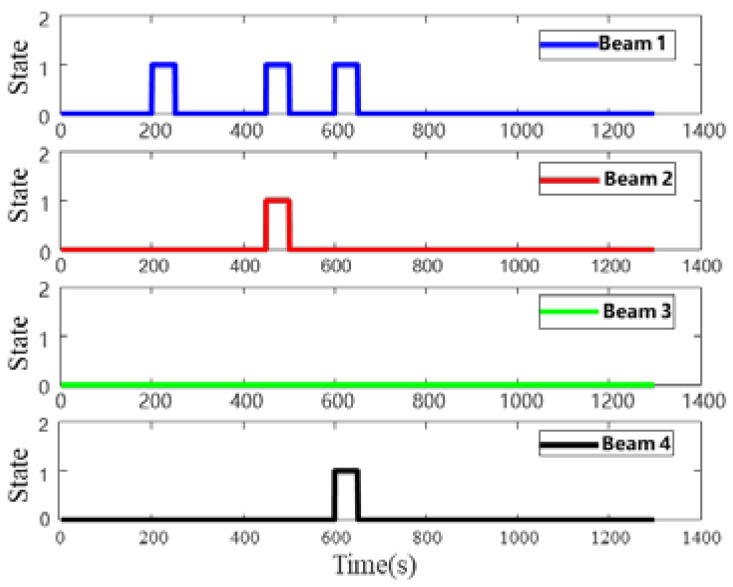
Beam state change process.

**Figure 10:**
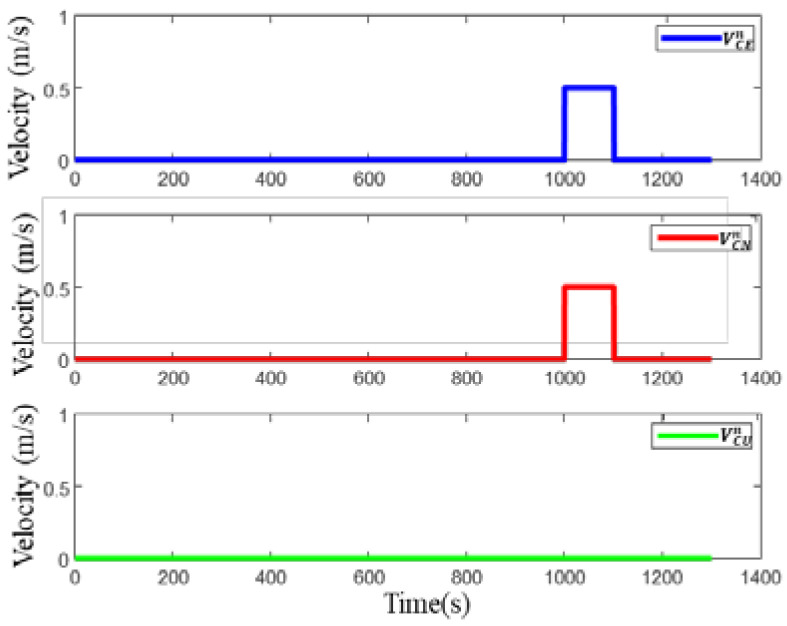
Variation of water flow velocity.

The impact of the external environment on DVL is shown in Figure 9 and Figure 10. The value 1 in Figure 9 indicates that the DVL beam is in an abnormal state, and the value 0 indicates that the DVL beam is in a normal state. In terms of the impact of water flow on DVL, the simulation is conducted on the impact of east flow velocity and north flow velocity, and the flow velocity is 0.5 m/s, as shown in Figure 10. There are four processes to simulate the abnormal situation of DVL shown in Figure 9 and Figure 10. The first phase is based on failure case 1 in DVL bottom-track measurement mode lasting for 50 s. The second phase is based on failure case 2 in DVL bottom-track measurement mode lasting for 50 s. The third phase is failure case 3 in the bottom-track measurement mode lasting for 50 s, and the fourth phase is the normal state, where the water flow affects the working status of DVL, lasting for 100 s.

**Figure 11:**
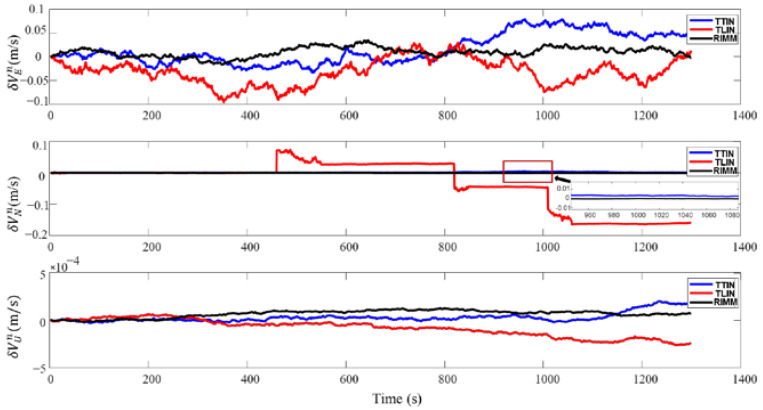
Velocity errors of three algorithms when DVL works in a complex environment.

**Figure 12:**
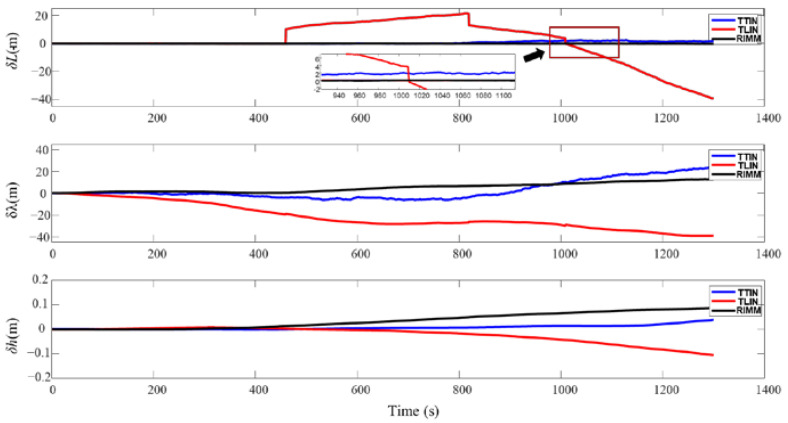
Position errors of three algorithms when DVL works in a complex environment.

The simulation results of DVL working in a complex environment are shown in Figure 11 and Figure 12. Since DVL has two kinds of beam information that cannot be used from 450 s to 500 s, the loosely integrated navigation (TLIN) can only be switched to the SINS and PS navigation modes. Furthermore, because of the small height change and the accurate height information proposed by PS, the velocity error and position error in the up direction are small. In the horizontal direction, the velocity and position of the AUV produce errors that cannot be quickly eliminated after DVL recovers to normal operation. Therefore, they continue to be accumulated in the AUV’s subsequent movement process. Table 5 shows that the maximum speed deviations of the east and north velocities are −0.0964 m/s and −0.1644 m/s, respectively. Table 6 also shows that the maximum deviations of latitude and longitude are −39.467 m and −39.125 m, respectively.

Compared with the loosely integrated navigation algorithm, the tightly integrated navigation (TTIN) algorithm and RIMM algorithm have higher stability. Figure 11 also shows that in the first three failure processes, both the tightly integrated navigation algorithm and RIMM algorithm can make full use of the effective information of the DVL beam. The application of Equations (37) and (38) ensures that the effective beam information is fully used to guarantee that the AUV is always in the SINS/DVL/PS integrated navigation mode. Due to the influence of water flow, the precision of the tightly integrated navigation algorithm only considering the beam starts to decrease. Table 5 and Table 6 show that the mean errors of the east velocity and the north velocity are 0.0156 m/s and 0.0007 m/s, respectively, and the maximum deviations of latitude and longitude are 2.234 m and 24.516 m, respectively.

Since the RIMM algorithm includes the water-track velocity measurement mode and bottom-track velocity measurement mode of DVL, the mean errors of the east velocity and the north velocity are 0.0098 m/s and −0.0002 m/s, respectively. The maximum deviations of latitude and longitude are 0.3427 m and 12.6886 m, respectively. Compared with the tightly integrated navigation algorithm, the mean errors of the east velocity and the north velocity decreased by 37.1% and 71.4%, respectively. The accuracy of the maximum deviations of latitude and longitude has also been improved by 84.6% and 48.2%, respectively. This is mainly because the RIMM algorithm not only has the advantage of the tightly integrated algorithm to make full use of the effective information of the DVL beam but also can make corresponding mode conversions according to the actual situation and use different models for combined filtering.

### 4.3. Comparison of RIMM and IMM Algorithms

**Figure 13:**
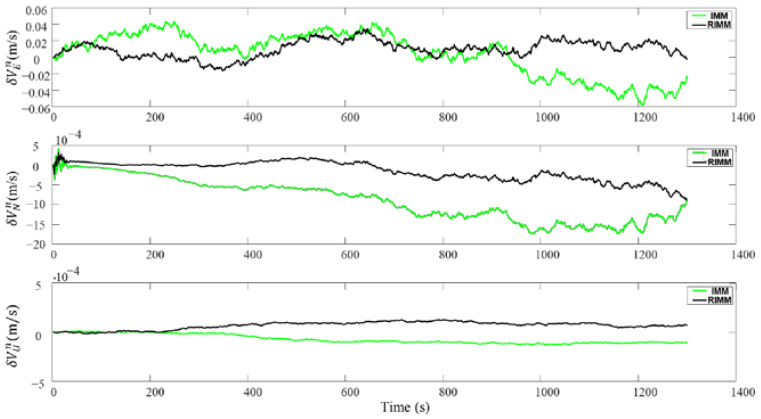
Velocity errors of RIMM and IMM.

**Figure 14:**
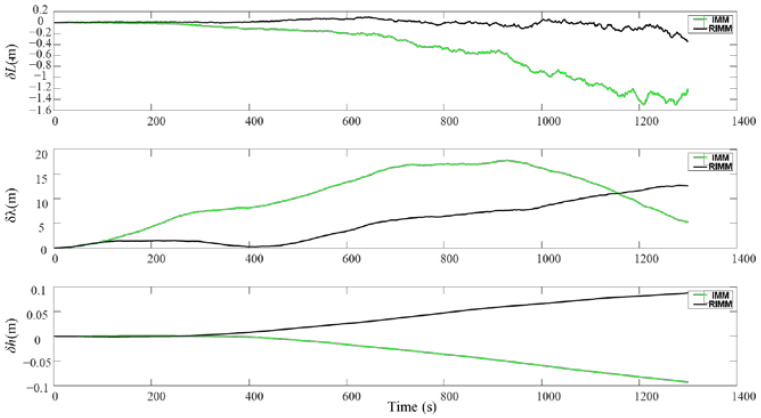
Position errors of RIMM and IMM.

Here, the traditional IMM algorithm is compared with the proposed RIMM algorithm. The DVL working environment in the simulation is consistent with that in Section 4.2. Through simulation, AUV velocity and position changes under the RIMM algorithm and the traditional IMM algorithm are shown in Figure 13 and Figure 14, where the results of the RIMM algorithm are shown by a black line, and the results of the IMM algorithm are shown by a green line. In the simulation process, due to the role of PS, the velocity and height in the up-change amplitude of the two algorithms are small. Therefore, this section mainly compares the differences between the two algorithms in plane velocity and position error optimization.

As shown in Figure 13 and Figure 14, in the first 200 s when DVL is operating normally, the traditional IMM algorithm and the proposed RIMM algorithm have the same positioning accuracy. After 200 s, the simulated environment begins to affect the DVL beam. These environmental factors mainly increase the observation noise of the corresponding DVL beam and affect the speed and position error of the underwater machine. Furthermore, SINS as the main positioning sensor is affected by the increase in the error at the auxiliary DVL sensor. Therefore, the cumulative errors cannot be effectively controlled and hence increase the velocity and position errors in both algorithms. However, compared with the traditional IMM algorithm, the instantaneous observation noise has less influence on the RIMM algorithm. Therefore, there are only slight changes in the speed and location errors.

In the simulation, the velocity and position error parameters of RIMM and IMM algorithms change, as shown in Table 7 and Table 8. The average and maximum position errors of the RIMM algorithm are smaller than those of the IMM algorithm. For the RMSE value, the east velocity and north velocity of the IMM algorithm are 0.0269 and 0.0013, respectively, and the east velocity and north velocity of the RIMM algorithm are 0.0084 and 0.0003, respectively. This shows that the RIMM algorithm is more stable than the IMM algorithm. In the case of position error, although the RIMM algorithm’s RMSE value of latitude error is 5.0236, which is higher than IMM’s RMSE value of longitude error of 2.8552, the maximum deviation of the RIMM algorithm’s longitude error during the whole simulation process is 10.5600 m, which is lower than IMM’s maximum deviation of longitude error of 17.7901 m. These indicate that although the longitude error greatly fluctuates, the accuracy remains acceptable.

**Figure 15:**
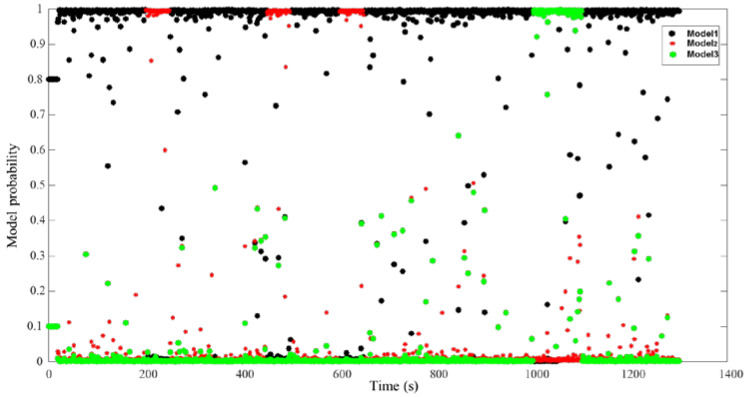
Model probability of IMM.

**Figure 16:**
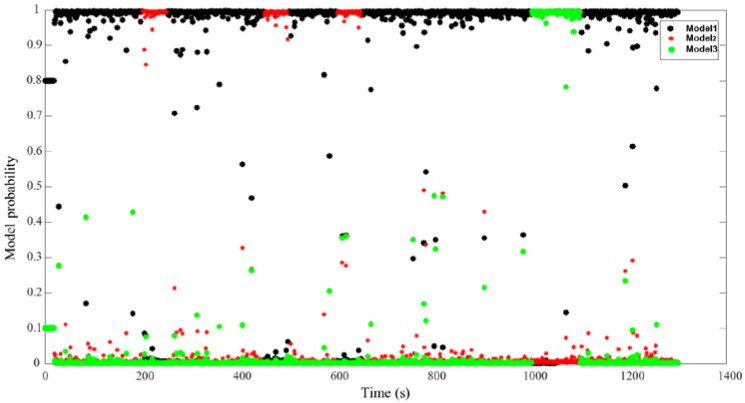
Model probability of RIMM.

Figure 15 and Figure 16 show the process of model probability change for the RIMM and IMM algorithms, which are the key factors determining filter precision. Model 1 shown by black points indicates that there is no external interference in the filtering process for DVL. Model 2 shown by red points indicates that DVL is filtered by the bottom-track measurement mode. Furthermore, Model 3 depicted by green points indicates that DVL is filtered by considering the influence of water flow. Figure 15 shows that the traditional IMM algorithm uses a fixed Markov transfer probability. When external noise is generated, the IMM algorithm cannot convert the probability quickly. This is the reason for the reduced navigation accuracy of the IMM algorithm.

Figure 16 also shows that the RIMM algorithm can be adaptively transformed to the corresponding model when the external environment of DVL is changed. When the external ambient noise of DVL disappears, RIMM can quickly convert to the corresponding model state. Compared with the traditional IMM algorithm, the adaptive Markov model probability change enables SINS and DVL integrated navigation to adapt to a variety of environmental changes, thus reducing errors in the navigation process and enhancing system stability.

## 5. Conclusions

In this paper, a novel tightly integrated navigation system of SINS, DVL, and PS was proposed for complex underwater environments. The tightly integrated navigation system is based on the DVL water-track velocity measurement model and the DVL bottom-track velocity measurement model. The RIMM algorithm was further proposed, which is based on a tightly integrated navigation system of SINS, DVL, and PS. This algorithm can detect the outliers and abnormal noises of DVL beams and use the variable Markov transition probability matrix to achieve fast conversion of different models. Simulating the motion process of the AUV in a complex environment, the results show that compared with the TLIN algorithm in terms of maximum deviation of latitude and longitude, the RIMM algorithm improves the accuracy by 39.1243 m and 26.4364 m, respectively. Compared with the TTIN algorithm, the RIMM algorithm also improves latitude and longitude accuracy by 1.8913 m and 11.8274 m, respectively. A comparison with IMM also shows that RIMM improves the accuracy of latitude and longitude by 1.1506 m and 7.2301 m, respectively. The proposed theoretical method effectively limits the impact of different environmental noises on the tightly integrated navigation system and improves the navigation accuracy of the entire navigation system.

Although the feasibility of the proposed algorithm is verified by simulation, it is still necessary to further verify the actual effect through experiments. In the future, we will verify the positioning efficiency and accuracy of the algorithm in the actual process through experiments and further consider the problem that the positioning accuracy may be reduced due to the interruption of inter-sensor communication in the actual process.

## Figures and Tables

**Table 1:** Main performance parameters of the sensors.

Sensors	Parameter	Value
Gyroscope	Biases drift	$0.02^{{}^{\circ}}/h$
	Random walk noise	$0.002^{{}^{\circ}}/\sqrt{h}$
Accelerometer	Biases drift	200 ug
	Random walk noise	$100\;\mathrm{ug}/\sqrt{\mathrm{Hz}}$
DVL	Biases drift	0.002 m/s
	Scale factor error	0.002
PS	Biases drift	0.005

**Table 2:** Velocity error parameters for the three algorithms when DVL working properly.

Algorithm	Content	${\delta V}_{E}^{n}\;(m/s)$	${\delta V}_{N}^{n}\;(m/s)$	${\delta V}_{U}^{n}\;(m/s)$
TTIN	maximum deviation	0.0182	0.0099	0.00030
	mean error	0.0004	−0.0004	0.00006
	RMSE	0.0075	0.0025	0.00015
TLIN	maximum deviation	−0.0157	−0.0066	0.00010
	mean error	−0.0033	−0.0014	0.00001
	RMSE	0.0063	0.0025	0.00004
RIMM	maximum deviation	0.0074	0.0024	0.00007
	mean error	0.0027	0.0008	0.00002
	RMSE	0.0037	0.0010	0.00003

**Table 3:** Position error parameters for the three algorithms when DVL working properly.

Algorithm	Content	δL (m)	δλ (m)	δh (m)
TTIN	maximum deviation	6.236	4.233	−0.084
	RMSE	0.1981	0.3133	0.0542
TLIN	maximum deviation	−4.803	−6.668	0.019
	RMSE	0.9117	0.9900	0.0093
RIMM	maximum deviation	1.7721	3.8828	−0.031
	RMSE	0.5119	1.2951	0.0194

**Table 4:** The failure modes of the DVL beam and operation modes of the integrated navigation system.

Failure Case	Failure Beam	Working Mode
1	Beam 1, Beam 2, Beam 3, or Beam 4	SINS, DVL, and PS
2	Beam 1 and Beam 2	SINS, DVL, and PS
3	Beam 1 and Beam 4	SINS, DVL, and PS
4	Beam 1 and Beam 3	SINS and PS
5	Beam 1, Beam 2, and Beam 3	SINS and PS
6	Beam 1, Beam 2, Beam 3, and Beam 4	SINS and PS

**Table 5:** Velocity error parameters for the three algorithms when DVL works in a complex environment.

Algorithm	Content	$\delta V_{E}^{n}\;(m/s)$	$\delta V_{N}^{n}\;(m/s)$	$\delta V_{U}^{n}\;(m/s)$
TTIN	maximum deviation	0.0792	0.0035	0.00020
	mean error	0.0156	0.0007	0.00002
	RMSE	0.0353	0.0013	0.00005
TLIN	maximum deviation	−0.0964	−0.1644	0.00026
	mean error	−0.0318	−0.0327	−0.00008
	RMSE	0.0419	0.0792	0.00012
RIMM	maximum deviation	0.0347	0.0009	0.00012
	mean error	0.0098	−0.0002	0.00006
	RMSE	0.0142	0.0003	0.00007

**Table 6:** Position error parameters for the three algorithms when DVL works in a complex environment.

Algorithm	Content	δL (m)	δλ (m)	δh (m)
TTIN	maximum deviation	2.234	24.516	0.037
	RMSE	0.5458	2.8552	0.0107
TLIN	maximum deviation	39.467	39.125	0.105
	RMSE	1.3033	21.5413	0.0380
RIMM	maximum deviation	0.3427	12.6886	0.087
	RMSE	0.0084	5.0236	0.0467

**Table 7:** Velocity error parameters for the two algorithms.

Algorithm	Content	$\delta V_{E}^{n}\;(m/s)$	$\delta V_{N}^{n}\;(m/s)$	$\delta V_{U}^{n}\;(m/s)$
IMM	maximum deviation	0.0585	−0.0017	−0.00013
	mean error	0.0044	−0.0009	−0.00007
	RMSE	0.0269	0.0013	0.00008
RIMM	maximum deviation	0.0347	0.0010	0.00012
	mean error	0.0098	−0.0002	0.00006
	RMSE	0.0142	0.0003	0.00007

**Table 8:** Position error parameters for the two algorithms.

Algorithm	Content	δL (m)	δλ (m)	δh (m)
IMM	maximum deviation	−1.4933	17.7901	0.091
	RMSE	0.4509	2.8552	0.0107
RIMM	maximum deviation	0.3427	10.5600	0.0426
	RMSE	0.0084	5.0236	0.0467

## Data Availability

Not applicable.
